# A modern conceptual framework for study and treatment of Meniere’s disease

**DOI:** 10.3389/fneur.2025.1607435

**Published:** 2025-05-16

**Authors:** Divya A. Chari, Arpan Bose, Kimberly Ramirez, Paula Robles-Bolivar, Kuei-You Lin, Amy F. Juliano, Steven D. Rauch, Andreas H. Eckhard

**Affiliations:** ^1^Department of Otolaryngology, Head and Neck Surgery, Massachusetts Eye and Ear, Harvard Medical School, Boston, MA, United States; ^2^Department of Otolaryngology, Head and Neck Surgery, UMASS Chan Medical School, Worcester, MA, United States; ^3^Eaton-Peabody Laboratories, Massachusetts Eye and Ear, Boston, MA, United States; ^4^Department of Otolaryngology, Head and Neck Surgery, Shin Kong Wu Ho-Su Memorial Hospital, Taipei, Taiwan; ^5^Department of Radiology, Massachusetts Eye and Ear, Harvard Medical School, Boston, MA, United States; ^6^Otopathology Laboratory, Massachusetts Eye and Ear, Boston, MA, United States

**Keywords:** Meniere’s disease, endolymphatic hydrops, endolymphatic sac dysfunction, endotypes, inner ear homeostasis, radiologic biomarkers

## Abstract

Prosper Meniere made his immortal contribution to the field of otology in 1861. At that time, all manner of “fits” were lumped together under the diagnosis of “apoplectiform cerebral congestion”—too much blood in the brain. His genius was to identify a specific subset of this heterogeneous pool whose cardinal symptoms, tinnitus, fluctuating progressive deafness, and episodic vertigo, were due to dysfunction of the inner ear. Seventy-seven years later, in 1938, Hallpike and Cairns in England and Yamakawa in Japan identified cochleosaccular endolymphatic hydrops (EH) as the histopathologic correlate of Meniere’s disease (MD). Over the 85 years since then, many theories to explain the symptoms of MD have come and gone. A consensus has slowly emerged that patients with this condition have a failure of inner ear homeostasis. The cause(s) of this homeostatic failure and the mechanism(s) by which this failure leads to fluctuating progressive sensorineural hearing loss and episodic vertigo has remained elusive. In the last few years, new techniques and findings in temporal bone histopathology and *in vivo* temporal bone imaging have yielded breakthroughs in this field. We are now recapitulating Meniere’s approach by taking the heterogeneous population of patients with MD and segregating them into specific subtypes based upon clinical phenotype. Salient clinical features include vestibular aqueduct and endolymphatic sac morphology, age at symptom onset, sex, and incidence of bilateral involvement. Furthermore, new imaging modalities enable unequivocal diagnosis of EH, transitioning MD from a “clinical” diagnosis to one based upon specific objective criteria. These breakthroughs have opened the door to genetic analyses, consideration of comorbid clinical disorders, especially migraine, and potential new treatments, and demand that we revisit all the various treatments that have been considered previously. They also demand new and more stringent criteria for any publication about this condition. In this paper we will review these new findings, discuss their immediate implications for clinical practice, and consider some of the most pressing research questions for near- and long-term address.

## 1 Meniere’s legacy: from stroke to inner ear pathology

Early references of vertigo are found in ancient Greek texts (1, 2). For centuries, vertigo was considered a cerebral symptom, akin to epileptic seizures and strokes, and fell under the broad and vague classification of “apoplectiform cerebral congestion.” This now-obsolete condition was managed with various bloodletting treatments, including leech therapy (3). In 1861, Prosper Meniere presented to the French Academy of Medicine, proposing that vertigo could originate from pathology in the inner ear rather than the brain (1). This radical idea, though initially met with skepticism, would become the cornerstone of Meniere’s legacy, laying the foundation for the idea that a group of patients can be segregated based on shared symptoms and a common underlying etiology.

Meniere’s legacy illustrates the power of reclassifying complex, heterogenous conditions into specific subgroups. We are now at another inflection point in the understanding of Meniere’s disease (MD), tasked with redefining patient classification in light of new insights into the underlying etiology(ies) of this condition. For the past 164 years, MD has been defined as a clinical syndrome, characterized by fluctuating and progressive sensorineural hearing loss, episodic vertigo, and aural fullness. The purpose of this manuscript is to revisit our evolving understanding of MD and chart the transition from a purely clinical diagnosis to one grounded in objective diagnostic criteria. This paper is not intended to be an exhaustive review of the MD literature, as has previously been published (4–6). Rather, it presents a concise synthesis of the authors’ recent findings placed within the context of the most relevant and influential prior work. We explore recent breakthroughs in histopathology, imaging, and genetic research that allow us to further stratify this heterogenous patient population into distinct subtypes. These developments mark a paradigm shift – rather than grouping all MD patients under one diagnostic umbrella, we now have the tools to segregate patients, an approach reminiscent of Meniere’s original pioneering work. This paper will examine these advances, their implications for clinical management, and the opportunities they present for improving diagnostic accuracy and therapeutic outcomes in this complex disorder.

## 2 The paradox: chasing a single cause in a multifaceted syndrome

MD has long been recognized as a clinically heterogenous disorder, with highly variable patterns of symptom onset, severity, duration, and progression (7). Some patients initially present with predominantly auditory symptoms, such as hearing loss or tinnitus, while others exhibit vestibular symptoms, including vertigo or imbalance. In many cases, one symptom domain may precede the other by years (8–10). For these reasons, establishing a clinical diagnosis remains challenging and is often made by exclusion, guided by international consensus criteria (11). In 1938, a parallel discovery of endolymphatic hydrops—distention of the endolymphatic space within the scala media—was made by Hallpike and Cairns in the United Kingdom and Yamakawa in Japan (12, 13). This observation quickly became the histopathologic hallmark of MD and was long regarded as the central pathophysiologic mechanism of definitive MD (14, 15). However, over time, doubts have emerged as to whether endolymphatic hydrops represents the primary driver of the disease or a secondary, epiphenomenal, process (16–18).

Efforts to categorize MD patients into distinct subtypes based on shared symptoms and/or etiology are not new. Early attempts focused on clinical presentations, such as “cochlear MD,” characterized by predominantly auditory symptoms of hearing loss and tinnitus, and “vestibular MD,” marked by episodic vertigo and imbalance, and disease laterality (19, 20). Others explored classification frameworks based on associated comorbidities, e.g., migraine (5, 21–23), allergy (24–26), vascular disorders (27, 28), autoimmune disease (29–32), and autonomic dysfunction (33, 34). Despite these efforts, therapeutic strategies often adhered to a so-called “60% rule,” in which approximately 60% of patients experienced symptomatic improvement (35). In retrospect, this incomplete treatment response likely reflects the inherent heterogeneity of the MD population. Without more precise stratification of MD patients, large scale efficacy in treatments may never be reached.

## 3 Reframing the paradigm: from endolymphatic Hydrops to endolymphatic sac deficiency

**Figure 1:**
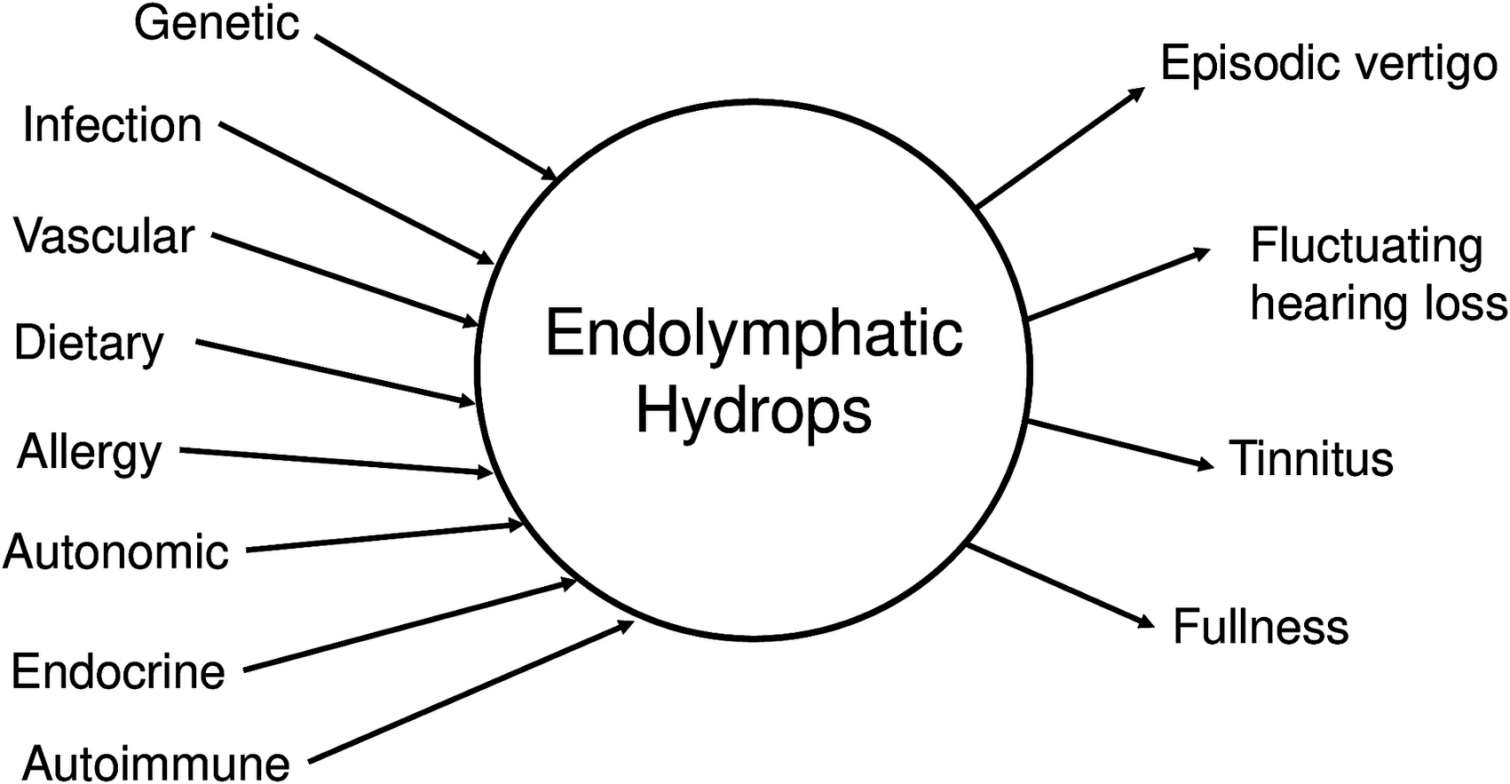
Central hypothesis for Meniere’s disease. Many possible etiologic factors can lead to endolymphatic hydrops, which in turn generates the clinical symptoms of MD.

According to dogma, endolymphatic hydrops is the pathological hallmark of MD, and the direct cause of the episodic hearing and balance symptoms that characterize it (12, 13). Figure 1 illustrates the classic model of the pathogenesis of MD (17), where multiple etiological factors converge to produce endolymphatic hydrops, which in turn generates the clinical symptoms of MD. This framework has dominated MD research and clinical decision making for decades, with endolymphatic hydrops thought to be both the final common pathway of MD and the cause for MD symptoms. The longstanding hypothesis is that a disturbed balance between endolymph fluid secretion and resorption caused a pathological increase in endolymph fluid volume and hydrostatic pressure, evoking mechanical stress on the endolymph-lining neuroepithelia by stretching, distorting and rupturing them, and thereby promoting progressive inner ear organ degeneration (8). This disease model was adopted, more than a century ago, from “fluid retention disorders,” (e.g., glaucoma and hydrocephalus), whose underlying pathophysiology was at the time well-established: a disturbed balance between organ fluid (vitreous humor, cerebrospinal fluid) production and drainage causes pathological fluid volume and pressure fluctuations within the eye and brain, respectively, eliciting visual and neurological symptoms during episodically recurring peak-pressure intervals (36, 37). The rationale for proposing an analogous pathophysiology for MD was initially based on the disease’s similar clinical presentations with recurring symptom episodes, and was further supported by the histopathological finding of enlarged endolymph fluid spaces in the inner ears from MD, which was interpreted as “endolymph hypertension.”

Although fluid hypertension has never been corroborated as the mechanism underlying the development of MD (17) various “fluid-draining” therapies, effective in resolving ocular/intracranial fluid hypertension and associated visual/neurological symptoms in glaucoma and hydrocephalus, were adopted for the treatment of MD. However, such therapies, medical and surgical, showed no demonstrable effects on episodic MD symptoms, or the overall disease progression (38–40). Paradoxically, despite their lack of proven efficacy, they remain the backbone of the first- and second-line standard of care in MD—mainly due to the general lack of effective (non-organ destructive) therapies. Taken together, the prevailing pathophysiological concept for MD was historically conceived based on *a priori* analogies that were in critical parts either never supported or were refuted by empirical data, and overall failed to spur the development of effective clinical therapies. From this, it is apparent that the basic requirements for launching successful drug discovery efforts, i.e., understanding the natural disease history, having knowledge about a potential molecular target, and its role in either the generation or amelioration of the disease state, are yet to be accomplished for MD.

**Figure 2:**
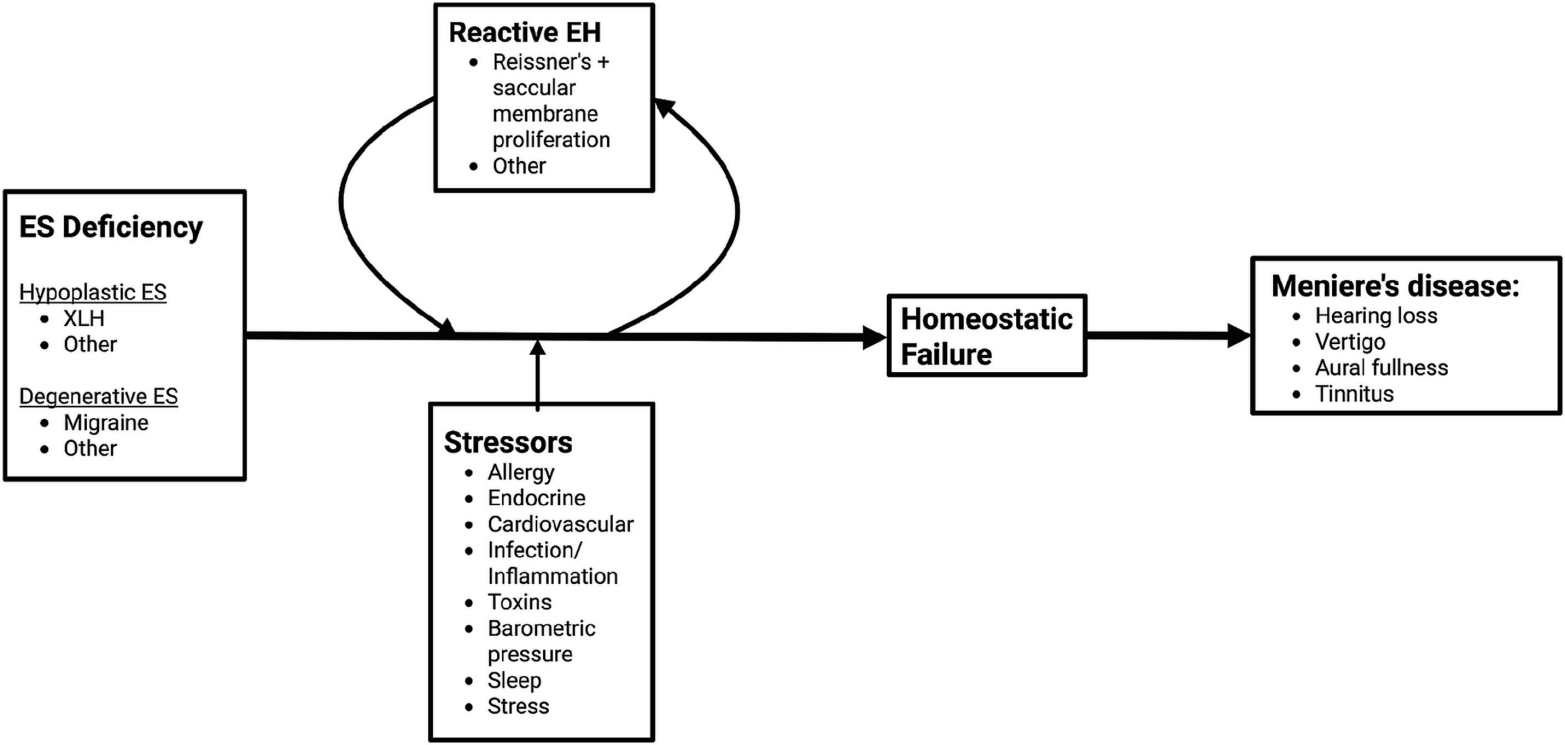
Novel framework for pathogenesis of Meniere’s disease. Endolymphatic sac (ES) deficiency may arise from ES developmental hypoplasia or ES degeneration. Reactive endolymphatic hydrops (EH), which stems from atypical proliferation of Reissner’s and the saccular membrane, along with stressors contribute to the development of inner ear homeostatic failure, which ultimately lead to the development of MD symptoms.

Much of the data and perspective presented in the following sections originate from the authors’ own investigations in histopathology, radiology, and clinical phenotyping. Building on these findings, we propose a novel conceptual framework to explain MD pathophysiology—Figure 2. This model places failure of inner ear homeostasis—particularly involving the endolymphatic sac (ES)—at the center of disease development, rather than endolymphatic hydrops. This failure may arise from two principal mechanisms: (1) primary deficiency of the ES, including developmental hypoplasia, or (2) secondary failure of the ES. Reactive endolymphatic hydrops due to proliferation-driven expansion of cochlear (Reissner’s membrane) and vestibular (primarily the saccular membrane) epithelia attempts to compensate primary ES homeostatic failure, whereas additional stressors (allergy, cardiovascular, toxins, barometric pressure, etc.) may contribute to ES homeostatic failure, ultimately giving rise to MD symptoms (41, 42). In this view, endolymphatic hydrops becomes not the cause, but an active cell-driven response to and a biomarker of disordered homeostasis within the membranous labyrinth. Recasting MD in this light opens the door to more precise subtyping and improved diagnostic criteria as well as targeted treatments that extend beyond hydrops management alone. Herein, we will describe the emerging evidence supporting this evolving model of MD pathophysiology.

### 3.1 Pathologies of the “distal” endolymphatic sac are universal among MD patients—etiologically diverse through convergent pathogenesis

Recent systematic temporal bone histopathological studies have consistently revealed distinct pathologies of the distal portion of the ES among MD patients (43, 44). In nearly every case, the distal ES exhibited abnormalities such as developmental hypoplasia, degeneration, or other disease-associated alterations. Radiological investigations have since established that these specific ES pathologies correlate with distinct clinical symptom profiles (phenotypes), offering a new perspective on the variable clinical presentation of MD (44–46). Longitudinal studies further demonstrated that inner ears with preexisting ES endotypes are predisposed to developing hydrops and subsequent MD symptoms, highlighting the critical role of the ES in disease pathogenesis (47, 48). Complementary molecular research in both human and animal models identified a mineralocorticoid-regulated transepithelial ion transport system within the distal ES (43, 49).

### 3.2 Reconceptualizing endolymphatic hydrops: a proactive stress response to counteract cellular loss and maintain inner ear homeostasis

Challenging the classical fluid-pressure hypothesis, advanced analyses of human temporal bone specimens have demonstrated that the previously reported epithelial ruptures are more accurately interpreted as histological artifacts. Instead, a consistent finding is the significant epithelial hyperplasia—up to seven-fold increases in cell numbers—in tissues affected by endolymphatic hydrops. These hyperplastic changes are observed across various disease stages and in both diffuse and focal forms of endolymphatic hydrops, suggesting that they are not the result of passive mechanical stretching but represent an active compensatory cellular response (41). Specifically, epithelial hyperplasia appears to compensate for cell loss in the distal ES, with newly formed cells expressing functional proteins crucial for maintaining fluid and ionic homeostasis. In other words, as the *functional* surface area of the ES epithelium decreases, the surface area of epithelia of the saccule and Reissner’s membrane increases (i.e., onset of epithelial hyperplasia). Initially adaptive in nature, this compensatory mechanism may eventually become maladaptive, contributing to progressive sensory deficits. Recognizing endolymphatic hydrops as an active response rather than merely a pressure-induced phenomenon shifts therapeutic strategies toward promoting beneficial epithelial growth and preventing maladaptive remodeling, opening new avenues for preserving hearing and balance in MD patients.

**Figure 3:**
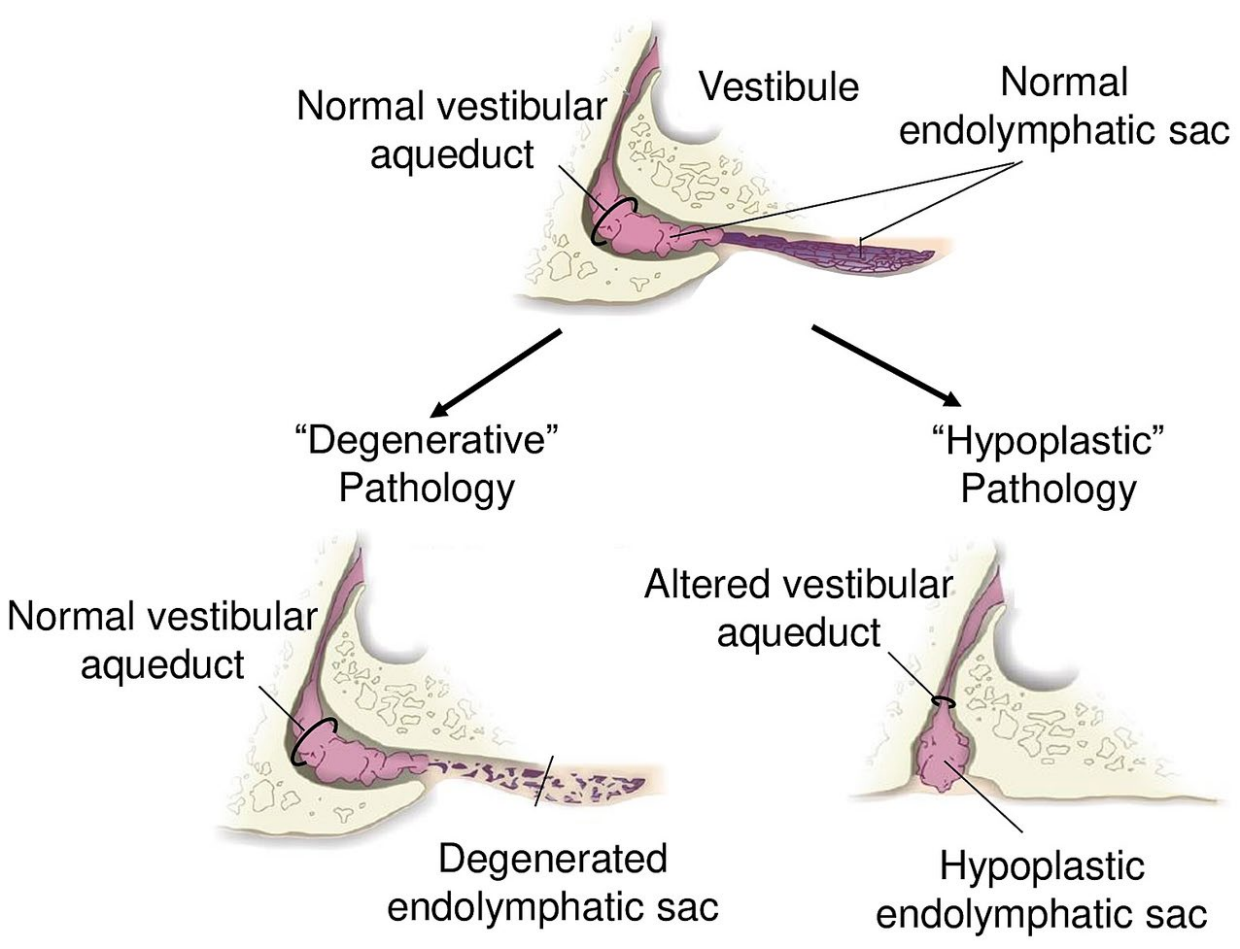
Schematic of endolymphatic sac pathologies. “Degenerative” pathology with a normal vestibular aqueduct and degenerated endolymphatic sac. “Hypoplastic” pathology with altered trajectory of the vestibular aqueduct and hypoplastic endolymphatic sac.

The recognition that MD is not a uniform entity, but rather comprises distinct pathological subtypes, has led to the introduction of the concept of clinical and pathophysiological “endotypes.” Recent radiologic and histopathological studies have provided compelling evidence that MD can be subdivided into at least two major endotypes based on the nature of distal ES pathology: a hypoplastic endotype (MD-hp) and a degenerative endotype (MD-dg, Figure 3). These endotypes not only differ in their underlying anatomical and histological alterations – developmental hypoplasia versus degeneration of the ES – but also show marked differences in clinical phenotype (44, 45, 47). Patients with the MD-hp endotype tend to present earlier in life, have a higher likelihood of bilateral disease, are more often male, and frequently report a positive family history of hearing loss and vertigo, suggesting a stronger genetic predisposition. In contrast, MD-dg patients typically exhibit later disease onset, more severe vestibular dysfunction, predominantly unilateral involvement, and are more likely to suffer from concurrent migraine. This endotype framework provides a unifying concept that links the previously discussed compensatory epithelial hyperplasia to distinct patterns of ES dysfunction (Figure 2). In both endotypes, compromised ES function appears to initiate a shared compensatory response—hyperplastic expansion of other endolymph-lining epithelia—aimed at stabilizing inner ear fluid homeostasis. However, the nature of the initiating ES pathology likely influences the dynamics, severity, and spatial distribution of this compensatory process, thereby contributing to the heterogeneous clinical manifestations of MD. Recognizing these endotypes holds immediate translational value: it offers an explanation for the variable clinical course, predicts different risks for bilateral progression, and may guide future therapeutic strategies. Tailoring interventions to endotype-specific mechanisms, rather than applying generalized pressure-reducing treatments, could improve outcomes and move MD management toward a precision medicine approach.

### 3.3 Clinical utility of endotyping: towards prognostic and therapeutic precision in MD

The emerging endotype concept has profound implications for the clinical management of MD. One of its most promising utilities lies in predicting disease laterality and progression. Radiological identification of vestibular aqueduct (VA) hypoplasia, a hallmark of the MD-hp endotype, can be readily achieved through computed tomography (CT) and magnetic resonance imaging (MRI), allowing clinicians to prospectively identify ears at risk. Studies have shown that VA hypoplasia is highly predictive of subsequent development of endolymphatic hydrops and symptomatic MD, even in initially asymptomatic ears, and is associated with a substantially increased risk for bilateral disease progression (44, 47). In contrast, patients with normal or degenerative ES and VA structures (MD-dg) tend to exhibit predominantly unilateral disease with a lower likelihood of bilateral conversion.

An increasingly compelling implication of the MD endotype framework is that MD-hp and MD-dg may not only differ in morphology and clinical course, but also in etiologic origin. The MD-hp endotype, characterized by ES developmental hypoplasia and a prematurely formed VA, may reflect a congenital malformation occurring during temporal bone development, raising the possibility of a genetic contribution to ES deficiency. By contrast, the MD-dg endotype, marked by degenerative changes in an otherwise normally developed ES and VA, appears to reflect an acquired pathology. Its strong association with migraine raises the possibility that migraine acts as a primary driver of ES degeneration and/or as a secondary stressor that exacerbates underlying instability in inner ear homeostasis once the ES is damaged or deficient.

This stratification has immediate practical benefits. For example, the detection of VA hypoplasia in one ear of a patient with unilateral MD may warrant more vigilant monitoring of the contralateral ear and early counseling regarding the risk of bilateral involvement. Furthermore, endotyping refines medical and surgical decision-making. Patients with the MD-hp endotype often present with smaller and more challenging anatomical landmarks during surgery (e.g., a hypoplastic VA and ES operculum), and display higher susceptibility to bilateral progression, making certain invasive procedures less favorable. Conversely, MD-dg patients, with more surgically accessible anatomy and lower risk for bilateral involvement, may be better candidates for traditional ablative or decompressing interventions. Indeed, this hypothesis may explain why endolymphatic sac surgery has been reported to achieve vertigo control in approximately two-thirds to three-quarters of MD patients (50, 51). In addition, MD-dg patients, with a higher concurrence of comorbid migraine, may benefit from an initial therapy trial of migraine preventative medications.

Beyond anatomy, endotyping opens the door to tailored therapeutic strategies aimed at modulating the specific pathological cascade involved—whether it be supporting residual ES function, enhancing compensatory epithelial plasticity, or preventing maladaptive remodeling. As diagnostic imaging becomes increasingly integrated into routine otological practice, endotyping may become a cornerstone in personalizing prognosis, follow-up intensity, and treatment selection for MD patients, ultimately contributing to more effective and patient-centered care.

## 4 The evolution of imaging in Meniere’s disease

### 4.1 Visualizing endolymphatic hydrops on imaging

For decades, the central question in imaging for MD was whether it would be possible to directly visualize endolymphatic hydrops—that is, to distinguish the endolymphatic space from the perilymphatic space *in vivo*. Major advances in MRI technology have now made that goal a reality. In 2005, Zou et al. demonstrated on MRI that a gadolinium-based contrast agent, delivered through the middle ear, could permeate the round window membrane and selectively enter the perilymphatic space after a time delay, without diffusing into the endolymphatic space. Importantly, they observed that intravenous administration of the same contrast agent also produced enhancement in the scala vestibuli and scala tympani of the cochlea (52). Naganawa et al. (53) showed that the 3D-FLAIR (Fluid Attenuated Inversion Recovery) MRI sequence could differentiate enhancing perilymph from non-enhancing endolymph in the cochlea following intravenous administration of gadolinium-based contrast, with an optimal imaging delay of 4 h. Then, in 2007, Nakashima, Naganawa et al. (54) achieved a landmark milestone by successfully visualizing endolymphatic hydrops in MD patients. Using intratympanic injection of contrast followed by a 3D-FLAIR MRI sequence after a one-day delay, they delineated the perilymphatic space via contrast enhancement, while showing that the endolymphatic space remained unenhanced, thereby enabling, for the first time, direct *in vivo* visualization of endolymphatic hydrops in the affected ears of MD patients.

In subsequent years, numerous studies investigated intratympanic and especially intravenous contrast administration routes, imaging sequences such as 3D-inversion recovery with real reconstruction (3D-REAL IR) in addition to 3D-FLAIR, variation in imaging parameters, and post-processing methods, all with the goal of optimizing visualization of the inner ear endolymph and perilymph spaces (55–62). At the same time, it was found that the degree of perilymph enhancement is increased in the setting of sensorineural hearing loss compared with normal-hearing individuals, and greater in MD-affected ears than in individuals with idiopathic sensorineural hearing loss, attributed to increased blood-perilymph barrier permeability (63). The combination of increased perilymph enhancement and endolymphatic hydrops was found to optimize sensitivity and specificity for MD (64, 65).

### 4.2 Beyond hydrops: imaging biomarkers for distal endolymphatic sac Endotypes

While the ability to visualize endolymphatic hydrops *in vivo* has been a landmark achievement, its utility remains largely confirmatory—to improve confidence in the clinical diagnosis of MD. The presence (or absence) of endolymphatic hydrops is unlikely to be specific enough to alter clinical management or predict disease course. As described earlier, imaging offers the potential to differentiate between distal ES endotypes. Combined histological and radiological studies have shown that the angular trajectory of the bony vestibular aqueduct (ATVA) can be used as a surrogate marker for the distal ES endotype, i.e., a more obtuse ATVA is highly predictive an MD-hp endotype, while a more acute ATVA correlates with the MD-dg endotype. (44) Interestingly, a wider ATVA is also linked to a thinner retrolabyrinthine bone, which carries clinical importance, since retrolabyrinthine bone thickness is more readily assessed on both CT and MRI compared to ATVA measurements. (46)

## 5 Revisiting the allergy hypothesis: chronic inflammation in MD

The connection between allergy and MD was first noted in 1923 when W.W. Duke reported resolution of MD-like symptoms in two patients after receiving epinephrine and avoiding allergic triggers (25). Cross-sectional studies have shown a higher prevalence of allergies in individuals with MD compared to the general population (24, 26, 66). Moreover, patients with MD exhibit elevated levels of IgE, immune complexes, interleukins, and autoantibodies when compared to control groups (26, 67). Dagli et al. (68) conducted a study in rabbits, revealing the presence of histamine receptor immunoreactivity within the endolymphatic sac. Since histamine plays a pivotal role in regulating allergic responses, some scientists argue that the existence of histamine receptors in this sac provides additional evidence supporting the connection between allergy and MD (69). While not approved for use in the United States, betahistine, a structural analog of histamine, is widely used in Europe and Asia as a treatment for MD (70, 71). The exact mechanism of action for betahistine remains unknown, but is believed to reduce the release of histamine and other neurotransmitters while improving microcirculation in the inner ear.

Derebery and Berlinger have proposed several hypotheses to connect allergy to MD: (1) the endolymphatic sac’s fenestrated blood supply may allow antigens to enter, leading to mast cell degranulation and inflammation, (2) circulating immune complexes could enter endolymphatic sac circulation and the stria vascularis, disrupting the normal fluid balance in the inner ear, (3) viral infections might exacerbate allergic symptoms by enhancing histamine release and damaging epithelial surfaces of the endolymphatic sac, triggering T-cell migration (24).

A recent study by Frejo et al. (72) provided evidence that allergy and autoinflammation contribute to persistent systemic inflammation in MD patients. Using cytokine profiling, the authors identified a distinct immunophenotype in approximately 25% of MD patients characterized by elevated IgE levels and specific Th2 cytokines, including IL-4, IL-5, IL-6, IL-10, and IL-13. This allergy-associated group showed sustained macrophage polarization, suggesting an ongoing type 2 immune response even in the absence of clinical allergy symptoms. Importantly, these immunologic patterns persisted over time, suggesting that subclinical inflammation may drive disease progression.

## 6 Genetic insights: a new frontier in Meniere’s disease subtyping

As we move toward a more refined classification of MD, genetic analysis offers a promising, but still developing, tool for subtyping patients. Much like histolopathologic and radiologic approaches have helped distinguish endotypes, genetics may offer a path to uncover shared mechanisms among a clinically heterogeneous group. Identifying genetic causes of MD has proven challenging due to its complex, multifactorial nature. Multiple research strategies have emerged, ranging from analysis of familial cases to stratifying patients based on shared phenotypes and endotypes. Below, we describe the key approaches, relevant findings, and ongoing challenges in this area of research.

### 6.1 Familial Meniere’s disease

Studying familial cases of MD has been an effective strategy for initial gene discovery (73–76). Several genes have been implicated in this manner, particularly those involved in inner ear development, tectorial membrane structure, and hair cell mechanotransduction. Mutations in genes such as *TECTA*, *OTOG*, and *STRC* suggest disruption in the mechanical coupling of the inner ear (75, 77, 78). *MYO7A*, *CDH23*, *PCDH15*, and *ADGRV1*, genes known for their role in mechanotransduction, have also been linked to familial MD (79). *HMX2*, *LSAMP*, *SEMA3D*, *DPT*, *PRKCB*, and *COCH*, have also emerged as potential candidates and are thought to play critical roles in the development and intracellular signaling pathways of the inner ear (80–84). These mutations exhibit a mix of dominant and recessive inheritance patterns, often with variable penetrance. The sheer number of implicated genes reflects both the complexity of the disorder and the likelihood that multiple genetic pathways may converge to produce a similar clinical syndrome. However, it remains unclear whether these mutations act early in development—predisposing the inner ear to structural vulnerabilities such as endolymphatic sac hypoplasia—or whether they exert their effects more directly at the level of sensory cell function, ultimately converging on the same symptomatic outcome through distinct pathogenic pathways.

### 6.2 Sporadic Meniere’s disease

While familial MD offers a foothold for genetic analysis, the majority of MD cases are sporadic, and therefore genetically far more difficult to interrogate. Traditional genome-wide association studies (GWAS) lack the statistical power to detect gene mutations due to the low disease prevalence of MD (~0.2%) (85) as causal variants would be expected to fall within the low to rare frequency range with a moderate to high impact (86). Instead, researchers have turned to variant burden analysis, a strategy that involves aggregating potential candidate variants based on the genes, pathways, or biological processes in which they are involved. By considering variant frequencies relevant to the disease, this approach increases the likelihood of identifying associations despite small sample sizes (86). Using this approach, several potential gene candidates have emerged. This approximation identified associations of sporadic MD with genes previously linked to SNHL (*GJB2, ESRRB*), ionic regulation of the endolymph (*SLC26A4, CLDN14*), and vestibular hypofunction in Usher syndrome (*USH1G*) (87). Variant burden associations have also been found in axonal guidance genes (*NTN4, NOX3*) (88). Of note, rare variants in familial MD genes (*FAM136A, DTNA, DPT*) were also detected in sporadic MD cases in South Korea (89). Nonetheless, similar to findings in familial MD, these gene associations in sporadic cases remain largely correlative, underscoring the need for functional validation in experimental models.

### 6.3 Genetic stratification of MD patients based on shared clinical phenotypes or endotypes

One promising strategy for elucidating the genetic architecture of MD involves stratifying patients by shared clinical features (phenotypes) or histological/radiologic characteristics (endotypes). This approach aims to reduce the heterogeneity of a broad population of MD patients and therefore increase the likelihood of identifying meaningful genetic associations. By narrowing patient cohorts based on disease expression, such as symptom severity, associated comorbidities, or histologic/radiologic endotypes, rare variants may be uncovered that may have been diluted or undetectable in a larger population. For example, Escalera-Balsera et al. (74) reported a subset of MD patients with severe tinnitus had rare mutations in the *ERBB3* and *AP4M1* genes, involved in Schwann cell maintenance and temporal bone development, respectively. Another avenue of research has explored immune-mediated subtypes. Some MD patients exhibit elevated autoantibodies, pro-inflammatory cytokines or IgE levels, suggesting a possible autoimmune or allergic component to MD in these individuals (90–95). Notably, genetic variants in immune-related genes have also been linked to MD susceptibility (73, 96–98). Finally, gene variants related to ion transportation, particularly sodium and potassium channels critical to inner ear homeostasis, have also been implicated in MD (73, 99, 100).

Cluster analyses have been used to identify clinical subgroups of Meniere’s disease based on disease parameters and associated comorbidities, i.e., migraine or autoimmune disorders. In a series of 153 patients with unilateral definite Meniere’s disease, Montes-Jovellar et al. (101) identified four distinct profiles of patients by considering age, auditory and vestibular assessments, and disability. Patients were characterized as “mildly active elderly,” “mildly active young,” “active compensated,” and “active uncompensated.” Frejo et al. (102) performed a two-step cluster analysis in 398 patients with bilateral Meniere’s disease and identified five distinct groups of patients. The largest group (Group 1), comprising 46% of patients, demonstrated sequential and progressive sensorineural hearing loss without a history of migraine or autoimmune comorbidities. Group 2, representing 17% of patients, included patients with concurrent bilateral onset of sensorineural hearing loss without migraine or autoimmune comorbidities. Group 3, with 13% of patients, consisted of patients with familial Meniere’s disease. Groups 4 and 5, each representing slightly over 10% of patients, encompassed those with a strong migraine history and patients with concurrent autoimmune disorders, respectively. In a subsequent study of 1,073 patients with unilateral Meniere’s disease from the same investigative group, the largest cohort, comprising over half of the patients, included individuals with sporadic Meniere’s disease without migraine or concurrent autoimmune disorders. The remaining groups consisted of patients with familial Meniere’s disease or comorbid conditions of migraine or autoimmune disease.

Recent work from our research group demonstrates compelling evidence of an epidemiological link between MD-hp and X-linked hypophosphatemia (XLH), a rare phosphate metabolism disorder caused by loss-of-function mutations in the *PHEX* gene located on the X chromosome that lead to hereditary rickets with skeletal and renal abnormalities (103). Given the independent prevalence rates of MD (104) and XLH (105), the expected probability of random co-occurrence would be in the millions. The observed co-occurrence in our cohort is several thousand times higher, suggesting a strong etiologic link between the disorders rather than chance occurrence. Notably, MD symptoms and VA hypoplasia were absent in all female XLH patients, but present in nearly all the male XLH patients. These findings highlight a male-specific pattern of MD in XLH patients, suggesting that complete loss of functional PHEX in XLH hemizygous male patients precipitates MD onset, whereas XLH heterozygous female patients retain partial PHEX function, which appears to confer protection for the inner ear.

## 7 Implications for future research

The story of Meniere’s disease began in 1861 with Prosper Meniere’s seminal insight—that the symptom triad of vertigo, hearing loss, and ear fullness could arise not from the cerebral pathology, but from inner ear dysfunction. Over the ensuing century and a half, this idea has evolved into a working model centered on endolymphatic hydrops—a concept that, while influential, ultimately proved insufficient to fully explain the variable disease progression and treatment response of MD patients.

Advances in histopathology, high-resolution imaging, and genomic analysis have converged to reshape our pathophysiologic understanding of MD—that MD is not a single disease, but a spectrum of disorders unified by a final common pathway resulting in failure of inner ear homeostasis driven by ES dysfunction. This paradigm shift holds profound implications. We can now identify biomarkers to differentiate subgroups of MD patients, allowing us to stratify risk and develop tailored therapies. Historically, MD management has followed a “top-down approach” in that patients were grouped under a broad diagnostic umbrella and therapeutic strategies were applied universally. The emerging “bottom-up approach,” grounded in shared clinical phenotypes and endotypes, offers a path toward precision medicine.

In this article, we propose a new framework for the understanding of the MD pathophysiology. One in which ES dysfunction (either primary or secondary via mechanisms such as migraine, allergy, toxins, stressors, genetic predisposition, etc.) gives rise to epithelial hyperplasia of the saccule and Reissner’s membrane, which in turn leads to homeostatic failure of the inner ear (Figure 2). While endolymphatic hydrops remains a key biomarker of MD, we argue that it is not the causative force, but rather a response to disrupted ES function.

Future research endeavors must aim to validate and integrate these evolving tools—histologic and radiologic biomarkers and genetic factors—into robust clinical frameworks. Large, multicenter studies with prospective designs will be essential to confirm the prognostic and therapeutic value of MD subtyping. Equally important will be the development of novel therapeutic agents tailored to specific MD endotypes—shifting the focus from symptom control to disease modification. In the spirit of Prosper Meniere’s original contribution, our challenge now is not simply to observe, but to classify with precision, and in doing so, transform how we understand and treat this complex disorder.
